# Supplementary material for: Out of East Asia: Early Warning of the Possible Invasion of the Important Bean Pest Stalk-Eyed Seed Bug Chauliops fallax (Heteroptera: Malcidae: Chauliopinae)
Source: Insects. 2023 May 1;14(5):433. doi: 10.3390/insects14050433 (PMC10231126; doi:10.3390/insects14050433)
Supplement: Supplementary file 1 [file insects-14-00433-s001.zip › insects-2342357-supplementary.pdf]

Supplementary Materials for

Out of East Asia: Early Warning of the Possible Invasion of the Important Bean Pest Stalk-Eyed Seed Bug *Chauliops fallax* (Heteroptera: Malcidae: Chauliopinae)

Yanfei Li [1,†], Shujing Wang [1,†], Juhong Chen [1,†], Kun Jiang [1], Jiayue Zhou [1], Runqi Zhu [1], Cuiqing Gao [2], Wenjun Bu [1,*] and Huaijun Xue [1,*]

[1] Institute of Entomology, College of Life Sciences, Nankai University, Tianjin 300071, China; liyanfei9412@163.com (Y.L.); wangsj@nankai.edu.cn (S.W.); 15181674153@163.com (J.C.); jiangkunnk@163.com (K.J.); 13836198804@163.com (J.Z.); zhurunqi1119@163.com (R.Z.)

[2] Co-Innovation Center for the Sustainable Forestry in Southern China, College of Forestry, Nanjing Forestry University, Nanjing 210037, China; cqgao@njfu.edu.cn

* Correspondence: wenjunbu@nankai.edu.cn (W.B.); xuehj@nankai.edu.cn (H.X.)

† These authors contributed equally to this paper.

Table S1 82 unique records of *Chauliops fallax* for ENM.

Species name	Longitude	Latitude
Chauliops fallax	96.78	28.71
Chauliops fallax	98.26	19.51
Chauliops fallax	98.92	28.46
Chauliops fallax	99.03	25.86
Chauliops fallax	100.45	21.96
Chauliops fallax	100.68	21.58
Chauliops fallax	100.80	22.01
Chauliops fallax	101.48	25.01
Chauliops fallax	102.23	29.91
Chauliops fallax	102.76	29.25
Chauliops fallax	102.84	29.88
Chauliops fallax	103.37	29.91
Chauliops fallax	103.39	29.57
Chauliops fallax	103.72	25.54
Chauliops fallax	104.57	31.53
Chauliops fallax	105.70	28.59
Chauliops fallax	107.18	27.96
Chauliops fallax	107.39	34.35
Chauliops fallax	107.86	28.37
Chauliops fallax	108.79	28.87
Chauliops fallax	109.04	33.79
Chauliops fallax	109.26	29.77
Chauliops fallax	109.30	32.93
Chauliops fallax	110.01	25.80
Chauliops fallax	110.31	25.06
Chauliops fallax	110.35	32.92
Chauliops fallax	110.74	32.06
Chauliops fallax	110.84	31.26
Chauliops fallax	111.01	26.40
Chauliops fallax	111.23	31.73
Chauliops fallax	112.94	28.19
Chauliops fallax	113.08	28.79
Chauliops fallax	113.39	32.36
Chauliops fallax	114.42	24.54
Chauliops fallax	114.48	31.10
Chauliops fallax	114.49	36.62
Chauliops fallax	114.62	28.53
Chauliops fallax	115.10	29.26
Chauliops fallax	115.69	31.19
Chauliops fallax	115.71	28.83
Chauliops fallax	115.74	26.89
Chauliops fallax	115.88	29.53

Chauliops fallax	116.02	30.99
Chauliops fallax	116.07	40.29
Chauliops fallax	116.41	39.88
Chauliops fallax	116.50	31.71
Chauliops fallax	116.75	36.55
Chauliops fallax	117.00	31.71
Chauliops fallax	117.14	27.70
Chauliops fallax	117.34	38.95
Chauliops fallax	117.41	40.05
Chauliops fallax	117.50	40.54
Chauliops fallax	117.57	30.05
Chauliops fallax	118.57	32.11
Chauliops fallax	118.67	28.32
Chauliops fallax	119.45	30.31
Chauliops fallax	119.76	31.26
Chauliops fallax	121.55	29.82
Chauliops fallax	126.34	36.76
Chauliops fallax	126.37	35.82
Chauliops fallax	126.44	37.74
Chauliops fallax	126.56	33.24
Chauliops fallax	126.70	35.44
Chauliops fallax	126.76	34.31
Chauliops fallax	127.00	37.42
Chauliops fallax	127.04	36.65
Chauliops fallax	127.27	35.00
Chauliops fallax	127.42	35.79
Chauliops fallax	127.75	34.68
Chauliops fallax	128.09	35.17
Chauliops fallax	128.45	37.28
Chauliops fallax	128.48	36.29
Chauliops fallax	129.18	35.62
Chauliops fallax	133.68	33.56
Chauliops fallax	134.24	35.50
Chauliops fallax	135.27	35.12
Chauliops fallax	137.00	36.62
Chauliops fallax	139.08	35.99
Chauliops fallax	139.16	35.33
Chauliops fallax	139.69	35.66
Chauliops fallax	140.18	35.97
Chauliops fallax	140.21	35.44

Table S2 Pairwise F_{ST} among populations

	AHFX	AHQM	HBWH	HNYL	JSNJ	JXJL	JXXY	SXQL	TJJX	ZJNB	ZJQZ	INDIA	GZSY	GZZY	SCLD	YNCX	YNQJ	THAI	XZCY
AHFX	0																		
AHQM	0.18667	0																	
HBWH	0.29486	-0.03152	0																
HNYL	0.81633	0.60123	0.38122	0															
JSNJ	-0.0207	0.11017	0.15079	0.40705	0														
JXJL	0.86207	0.72716	0.61093	-0.3211	0.59281	0													
JXXY	0.86068	0.6875	0.53871	0	0.53105	-0.07784	0												
SXQL	0.03226	0.1501	0.21185	0.7913	-0.05131	0.87566	0.87603	0											
TJJX	0.08657	0.18388	0.23636	0.71429	0.02647	0.82198	0.80263	0.04846	0										
ZJNB	0	0.11375	0.17273	0.86813	-0.08216	0.90756	0.93213	-0.01961	0.01493	0									
ZJQZ	0	0.17919	0.26715	0.83784	0.0013	0.88458	0.88971	0.01935	-0.05	0	0								
INDIA	0.64706	0.38095	0.22222	1	0.01333	0.92857	1	0.6	0.55	0.71429	0.71429	0							
GZSY	0.91781	0.80741	0.71717	1	0.71318	0.97561	1	0.88235	0.85	0.92	0.91667	1	0						
GZZY	0.86877	0.82571	0.79586	0.86052	0.77601	0.90567	0.89202	0.84622	0.83333	0.84789	0.85747	0.81667	-0.8333	0					
SCLD	0.93232	0.86385	0.84997	0.9803	0.81221	0.97715	0.98483	0.93813	0.90703	0.9551	0.93939	0.96721	0.96721	0.86339	0				
YNCX	0.92857	0.87552	0.85035	0.94824	0.82757	0.9593	0.96106	0.92376	0.90423	0.93403	0.92907	0.93421	-0.25	0.18009	0.9438	0			
YNQJ	0.9589	0.8985	0.87689	1	0.84917	0.9878	1	0.96733	0.94156	0.98377	0.96772	1	0	0.06955	0.98266	0.375	0		
THAI	0.92683	0.82667	0.74429	1	0.7457	0.97802	1	0.89474	0.86667	0.92857	0.92593	1	1	0.84058	0.97802	0.94118	1	0	
XZCY	0.92986	0.836	0.78039	1	0.76241	0.98228	1	0.91273	0.88679	0.9469	0.93407	1	1	0.91464	0.9863	0.96822	1	1	0

Table S3 Pairwise F_{ST} among four groups

	EA	WE	XZ	TL
EA	0			
WE	0.7047	0		
XZ	0.77586	0.81682	0	
TL	0.7672	0.68597	1	0

Table S4 Marginal likelihood of optimal partition of different K value

k	Log (marginal likelihood) of optimal partition
1	-1140.2501
2	-753.6297
3	-691.1481
4	-661.0483
5	-646.5017
6	-644.2616
7	-686.7286
8	-735.1608
9	-786.1432
10	-842.6708

Table S5 SAMOVA results

K value	FSC	FCT	FST
2	0.70307	0.64162	0.89359
3	0.58426	0.72753	0.88672
4	0.42548	0.77311	0.86965
5	0.27115	0.82098	0.86952
6	0.26417	0.82125	0.86847
7	0.01888	0.85098	0.8538
8	-0.01043	0.85093	0.84938
9	-0.02427	0.85128	0.84767
10	-0.07023	0.85124	0.84079

Table S6 MaxEnt results with percentage contribution (C) and permutation importance (P) of each variable predictor (with AUC values) for *Chauliops fallax*

Replicates	Training AUC	Test AUC
Repeat 1	0.8311	0.8509
Repeat 2	0.8234	0.8392
Repeat 3	0.7936	0.8189
Repeat 4	0.8338	0.908
Repeat 5	0.7887	0.8233
Repeat 6	0.7632	0.7614
Repeat 7	0.8274	0.7956
Repeat 8	0.8293	0.8428

Repeat 9	0.8371	0.8249
Repeat 10	0.8663	0.7692
Repeat 11	0.8589	0.7916
Repeat 12	0.8294	0.8048
Repeat 13	0.8553	0.7616
Repeat 14	0.8317	0.8085
Repeat 15	0.8416	0.7908
Average	0.8274	0.8128

Table S7 Predicted coverage area (km^2) of the potential distribution of *Chauliops fallax* under the present climatic conditions.

Suitability	Africa	Asia	Europe	North America	Oceania	South America	Total
Poor	1.03×10^6	1.86×10^6	3.01×10^6	2.58×10^6	1.22×10^6	1.97×10^6	1.17×10^7
Moderate	9.52×10^4	4.84×10^5	5.40×10^4	1.09×10^6	2.70×10^4	1.60×10^5	1.91×10^6
High	4.27×10^4	2.53×10^6	2.42×10^4	6.59×10^5	1.07×10^3	1.63×10^3	3.26×10^6
Total	1.17×10^6	4.88×10^6	3.09×10^6	4.33×10^6	1.24×10^6	2.13×10^6	1.68×10^7

Table S8 Predicted coverage area change (km^2) of the potential distribution of *Chauliops fallax* under the future climatic compared to present climate condition

Projected scenarios	Suitability	Africa	Asia	Europe	North America	Oceania	South America	Total
2060_CAN_SSP126	Poor	6.55×10^5	7.83×10^4	-2.10×10^6	-1.23×10^6	7.95×10^5	7.44×10^5	-1.06×10^6
	Moderate	5.27×10^4	-2.88×10^5	-8.80×10^4	5.75×10^5	2.40×10^4	1.56×10^5	4.32×10^5
	High	2.50×10^4	8.94×10^5	-5.56×10^4	1.85×10^5	1.04×10^3	8.17×10^2	1.05×10^6
2060_CAN_SSP585	Poor	5.63×10^5	-1.13×10^6	-2.53×10^6	-1.61×10^6	6.06×10^5	6.93×10^5	-3.41×10^6
	Moderate	3.63×10^4	-2.14×10^5	-9.51×10^4	2.90×10^5	6.98×10^3	1.08×10^4	3.44×10^4
	High	1.96×10^4	8.04×10^5	-1.15×10^5	-1.08×10^6	3.19×10^2	-2.40×10^4	-4.00×10^5
2060_IPSL_SSP126	Poor	4.66×10^5	-5.33×10^5	-1.66×10^6	-6.30×10^5	4.92×10^5	5.99×10^5	-1.27×10^6
	Moderate	1.99×10^4	-1.64×10^5	-1.18×10^5	5.29×10^5	1.34×10^4	2.13×10^4	3.01×10^5
	High	-1.66×10^4	4.65×10^5	-8.35×10^4	-9.31×10^5	1.00×10^3	7.58×10^2	-5.65×10^5
2060_IPSL_SSP585	Poor	7.79×10^5	1.16×10^5	-1.57×10^6	-8.98×10^5	7.89×10^5	7.82×10^5	2.50×10^3
	Moderate	6.02×10^4	-2.03×10^5	-4.19×10^4	2.39×10^5	-1.20×10^4	5.68×10^4	9.96×10^4
	High	2.19×10^4	6.65×10^5	-1.08×10^5	-4.26×10^5	-2.33×10^3	1.55×10^3	1.52×10^5
2060_MPI_SSP126	Poor	6.98×10^5	-6.03×10^4	-1.02×10^6	1.62×10^5	6.12×10^5	7.43×10^5	1.14×10^6
	Moderate	9.17×10^4	-6.09×10^4	-2.71×10^4	5.61×10^4	2.53×10^4	1.60×10^5	2.45×10^5
	High	4.25×10^4	4.97×10^5	-1.09×10^4	-2.63×10^3	1.07×10^3	1.63×10^3	5.29×10^5
2060_MPI_SSP585	Poor	6.51×10^5	3.83×10^5	6.61×10^5	-1.89×10^5	8.74×10^5	1.02×10^6	3.40×10^6
	Moderate	4.11×10^4	-3.30×10^4	2.99×10^4	5.97×10^5	2.68×10^4	1.59×10^5	8.21×10^5
	High	3.72×10^4	6.48×10^5	1.22×10^4	5.90×10^4	1.07×10^3	1.63×10^3	7.59×10^5
2100_CAN_SSP126	Poor	-9.50×10^2	-1.79×10^6	-2.25×10^6	-1.01×10^6	-1.88×10^5	4.13×10^5	-4.83×10^6
	Moderate	-3.41×10^4	-1.72×10^5	-4.14×10^5	3.74×10^5	-4.48×10^4	-1.81×10^5	-4.71×10^5

	High	-1.34×10^5	3.14×10^5	-1.66×10^5	-1.51×10^6	-1.64×10^4	-1.33×10^5	-1.65×10^6
2100_CAN_SSP585	Poor	9.18×10^5	-2.22×10^6	-4.92×10^6	-5.99×10^6	9.89×10^5	1.47×10^6	-9.75×10^6
	Moderate	7.92×10^4	-3.85×10^4	-3.24×10^5	-3.32×10^5	2.53×10^4	1.58×10^5	-4.33×10^5
	High	4.14×10^4	1.18×10^6	-8.78×10^4	1.09×10^5	1.07×10^3	1.55×10^3	1.25×10^6
2100_IPSL_SSP126	Poor	7.86×10^5	4.03×10^5	-9.63×10^5	-4.82×10^5	9.02×10^5	1.07×10^6	1.71×10^6
	Moderate	6.27×10^4	-1.20×10^5	-3.26×10^4	3.31×10^5	2.62×10^4	1.21×10^5	3.88×10^5
	High	2.42×10^4	4.70×10^5	-5.06×10^4	-8.94×10^4	1.02×10^3	1.13×10^3	3.56×10^5
2100_IPSL_SSP585	Poor	5.71×10^5	-5.20×10^6	-7.05×10^6	-7.07×10^6	7.46×10^5	5.43×10^5	-1.75×10^7
	Moderate	6.21×10^4	-2.47×10^5	-5.91×10^5	-8.90×10^4	1.85×10^3	1.15×10^5	-7.48×10^5
	High	3.90×10^4	1.35×10^6	-2.33×10^5	-1.73×10^6	-8.11×10^1	-1.39×10^3	-5.75×10^5
2100_MPI_SSP126	Poor	6.98×10^5	3.63×10^5	-5.71×10^5	-2.71×10^5	8.54×10^5	1.17×10^6	2.24×10^6
	Moderate	7.05×10^4	-9.48×10^4	5.17×10^3	3.14×10^5	2.70×10^4	1.60×10^5	4.82×10^5
	High	4.21×10^4	6.91×10^5	1.02×10^4	5.50×10^5	1.07×10^3	1.63×10^3	1.30×10^6
2100_MPI_SSP585	Poor	6.86×10^5	-1.50×10^6	-2.57×10^6	-1.22×10^6	9.54×10^5	1.03×10^6	-2.61×10^6
	Moderate	5.56×10^4	-1.24×10^5	-1.45×10^3	4.81×10^5	2.69×10^4	1.60×10^5	5.98×10^5
	High	2.80×10^4	8.51×10^5	1.17×10^4	-7.19×10^5	1.07×10^3	1.63×10^3	1.75×10^5

Table S9 Predicted the overlap coverage area (km^2) between the soybean planted area and potential distribution of *Chauliops fallax* in the future compared to present climate condition.

Projected scenarios	Suitability	Africa	Asia	Europe	North America	Oceania	South America	Total
2060_CAN_SSP126	Poor	-5.70×10^{4}	1.46×10^{5}	9.74×10^{5}	8.65×10^{5}	-1.23×10^{5}	-3.98×10^{5}	1.41×10^{6}
	Moderate	-3.12×10^{4}	2.03×10^{5}	3.00×10^{4}	-2.96×10^{5}	-6.67×10^{3}	-1.11×10^{5}	-2.11×10^{5}
	High	-2.24×10^{4}	-7.53×10^{5}	2.02×10^{4}	-1.20×10^{5}	-7.48×10^{1}	-1.55×10^{2}	-8.75×10^{5}
2060_CAN_SSP585	Poor	-7.28×10^{4}	5.67×10^{5}	1.23×10^{6}	3.46×10^{5}	-7.71×10^{4}	-4.20×10^{5}	1.58×10^{6}
	Moderate	-2.45×10^{4}	1.71×10^{5}	3.72×10^{4}	-2.18×10^{5}	-2.12×10^{3}	4.73×10^{3}	-3.18×10^{4}
	High	-1.93×10^{4}	-7.15×10^{5}	3.64×10^{4}	9.75×10^{5}	-1.95×10^{1}	1.86×10^{4}	2.95×10^{5}
2060_IPSL_SSP126	Poor	-7.55×10^{4}	2.87×10^{5}	8.59×10^{5}	3.08×10^{5}	-5.74×10^{4}	-3.09×10^{5}	1.01×10^{6}
	Moderate	-1.00×10^{4}	1.05×10^{5}	5.68×10^{4}	-3.84×10^{5}	-3.50×10^{3}	-9.39×10^{3}	-2.45×10^{5}
	High	1.02×10^{4}	-4.07×10^{5}	3.73×10^{4}	8.30×10^{5}	-7.48×10^{1}	-1.36×10^{2}	4.70×10^{5}
2060_IPSL_SSP585	Poor	-9.81×10^{4}	9.28×10^{4}	6.90×10^{5}	2.90×10^{5}	-1.16×10^{5}	-4.17×10^{5}	4.42×10^{5}
	Moderate	-3.89×10^{4}	1.08×10^{5}	7.27×10^{3}	-1.11×10^{5}	-3.66×10^{3}	-3.44×10^{4}	-7.26×10^{4}
	High	-1.97×10^{4}	-5.56×10^{5}	$2.91x10^{4}$	4.21×10^{5}	-1.22×10^{0}	-5.52×10^{2}	-1.26×10^{5}
2060_MPI_SSP126	Poor	-4.49×10^{4}	1.41×10^{5}	4.70×10^{5}	7.74×10^{4}	-1.25×10^{5}	-4.87×10^{5}	3.16×10^{4}
	Moderate	-5.98×10^{4}	4.81×10^{4}	8.73×10^{3}	4.41×10^{4}	-7.69×10^{3}	-1.13×10^{5}	-8.00×10^{4}
	High	-3.98×10^{4}	-4.39×10^{5}	3.50×10^{3}	5.69×10^{4}	-7.48×10^{1}	-5.72×10^{2}	-4.19×10^{5}
2060_MPI_SSP585	Poor	-5.98×10^{4}	-3.27×10^{4}	-7.19×10^{4}	4.72×10^{5}	-6.63×10^{4}	-3.57×10^{5}	-1.16×10^{5}
	Moderate	-1.79×10^{4}	1.31×10^{4}	-1.44×10^{4}	-3.24×10^{5}	-7.30×10^{3}	-1.14×10^{5}	-4.64×10^{5}
	High	-3.46×10^{4}	-5.58×10^{5}	-4.05×10^{3}	1.16×10^{4}	-7.48×10^{1}	-5.72×10^{2}	-5.86×10^{5}
2100_CAN_SSP126	Poor	-1.95×10^{4}	6.60×10^{5}	9.76×10^{5}	1.00×10^{5}	3.77×10^{4}	-3.31×10^{5}	1.42×10^{6}
	Moderate	-1.45×10^{4}	1.35×10^{5}	2.12×10^{5}	-3.08×10^{5}	1.06×10^{4}	1.34×10^{5}	1.69×10^{5}
	High	7.83×10^{4}	-3.17×10^{5}	8.40×10^{4}	1.21×10^{6}	6.11×10^{3}	1.03×10^{5}	1.16×10^{6}
2100_CAN_SSP585	Poor	-1.66×10^{5}	7.73×10^{5}	1.39×10^{6}	1.35×10^{6}	-1.59×10^{5}	-7.48×10^{5}	2.44×10^{6}
	Moderate	-5.95×10^{4}	-6.35×10^{4}	2.55×10^{4}	1.23×10^{5}	-7.34×10^{3}	-1.12×10^{5}	-9.38×10^{4}

	High	-3.94×10^{4}	-1.04×10^{6}	1.25×10^{4}	-1.51×10^{5}	-7.48×10^{1}	-5.33×10^{2}	-1.22×10^{6}
2100_IPSL_SSP126	Poor	-1.07×10^{5}	-1.40×10^{5}	5.70×10^{5}	3.56×10^{5}	-1.29×10^{5}	-4.46×10^{5}	1.03×10^{5}
	Moderate	-3.90×10^{4}	7.50×10^{4}	5.77×10^{3}	-1.09×10^{5}	-7.62×10^{3}	-8.30×10^{4}	-1.58×10^{5}
	High	-2.18×10^{4}	-3.89×10^{5}	1.37×10^{4}	1.41×10^{5}	-7.48×10^{1}	-2.75×10^{2}	-2.57×10^{5}
2100_IPSL_SSP585	Poor	-9.64×10^{4}	2.26×10^{6}	2.11×10^{6}	9.97×10^{5}	-9.75×10^{4}	-5.06×10^{5}	4.66×10^{6}
	Moderate	-4.45×10^{4}	1.24×10^{5}	9.07×10^{4}	-2.36×10^{5}	-7.45×10^{2}	-7.47×10^{4}	-1.42×10^{5}
	High	-3.89×10^{4}	-1.19×10^{6}	4.08×10^{4}	1.04×10^{6}	4.59×10^{2}	2.08×10^{3}	-1.51×10^{5}
2100_MPI_SSP126	Poor	-6.87×10^{4}	-7.52×10^{4}	2.72×10^{5}	4.81×10^{5}	-1.26×10^{5}	-5.63×10^{5}	-7.99×10^{4}
	Moderate	-3.90×10^{4}	7.39×10^{4}	-7.23×10^{3}	-7.28×10^{4}	-7.71×10^{3}	-1.14×10^{5}	-1.66×10^{5}
	High	-3.95×10^{4}	-5.96×10^{5}	-4.00×10^{3}	-4.15×10^{5}	-7.48×10^{1}	-5.72×10^{2}	-1.05×10^{6}
2100_MPI_SSP585	Poor	-7.03×10^{4}	7.18×10^{5}	9.82×10^{5}	3.29×10^{5}	-1.36×10^{5}	-6.20×10^{5}	1.20×10^{6}
	Moderate	-3.53×10^{4}	6.88×10^{4}	-5.10×10^{3}	-2.75×10^{5}	-7.69×10^{3}	-1.14×10^{5}	-3.68×10^{5}
	High	-2.54×10^{4}	-7.40×10^{5}	-5.36×10^{3}	6.62×10^{5}	-7.48×10^{1}	-5.72×10^{2}	-1.09×10^{5}

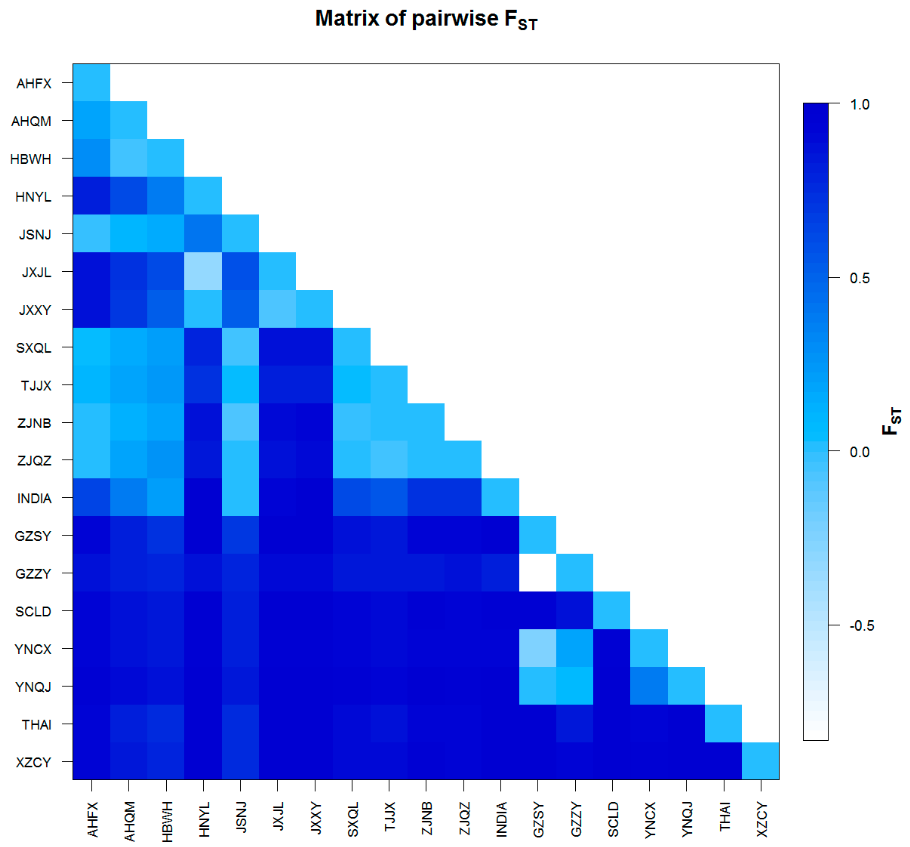

Figure S1 Pairwise F_{ST} among populations

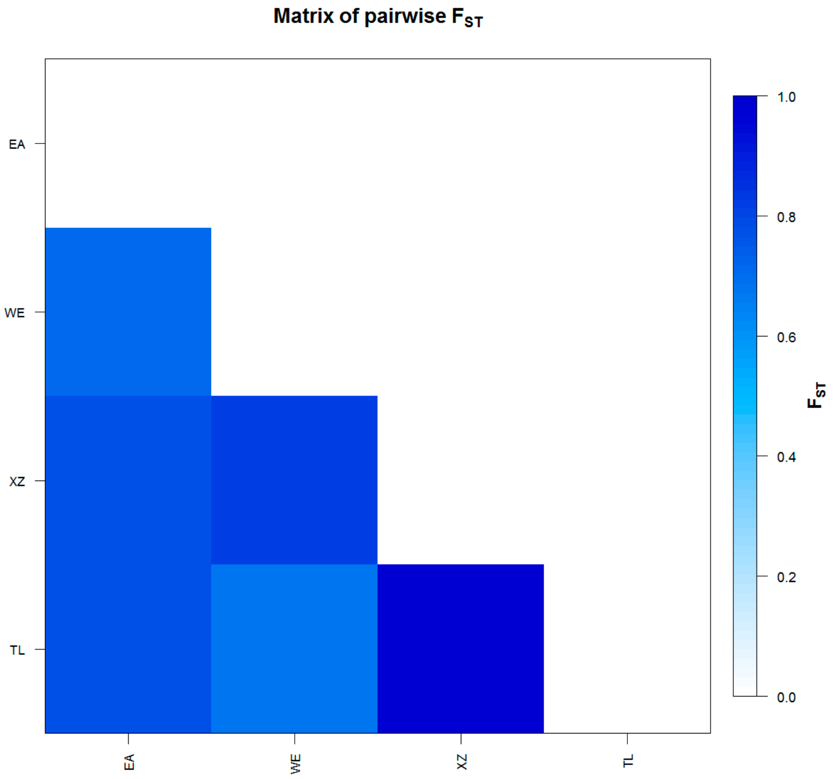

Figure S2 Pairwise F_{ST} among four groups

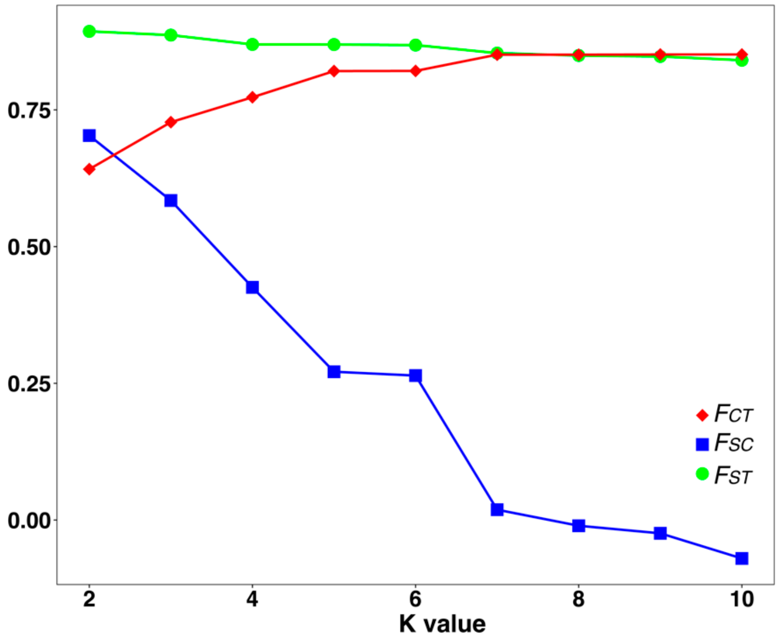

Figure S3 SAMOVA clustering, ordinate represents the values of F-statistics (blue line: differences between populations within groups, FSC; green line: differences within populations, FST; red line: differences between groups, FCT); and (c) barrier clustering.

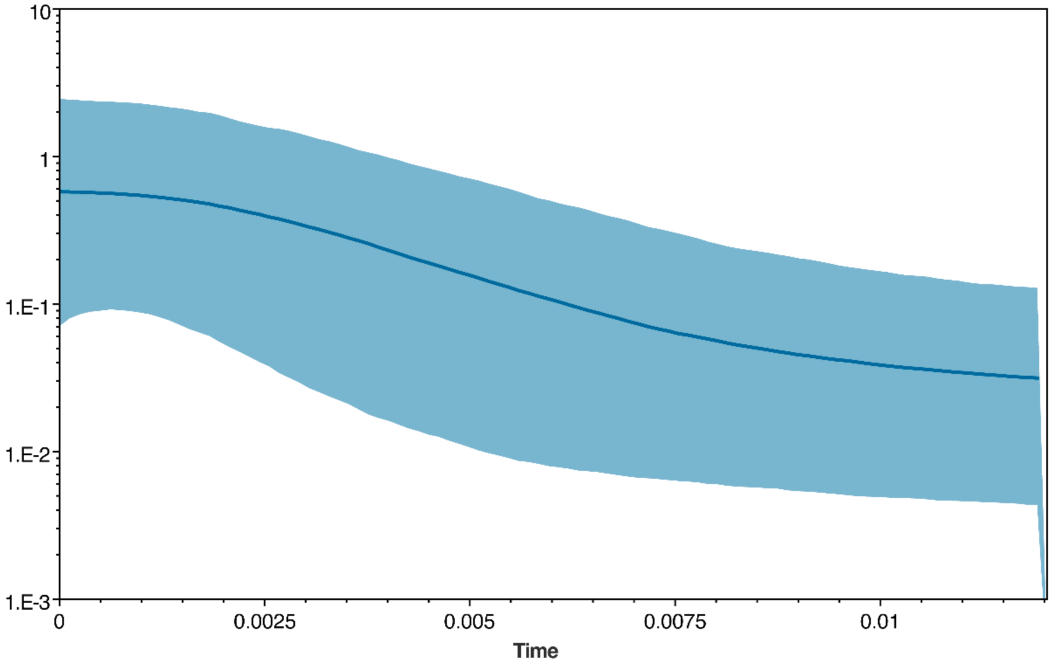

Figure S4 Historical demographic trends of EA group represented by Bayesian skyline plot (BSP). The time scale before present is shown on the x-axis, and the estimated effective population size appears on the y-axis. Estimates of means are joined by a solid line, and the shaded range delineates the 95% HPD limits.

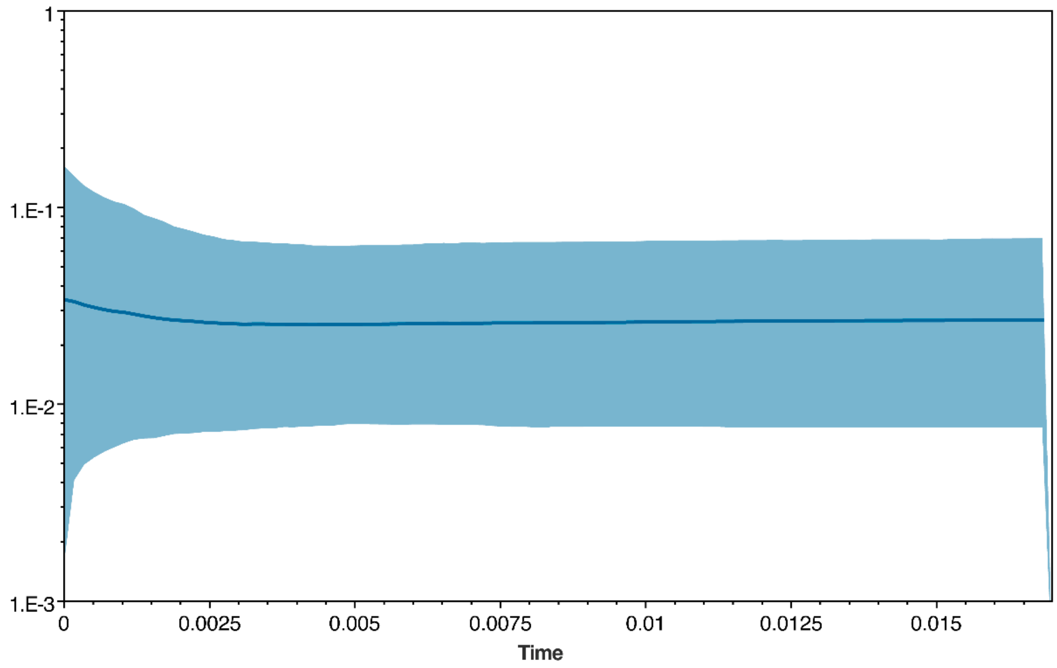

Figure S5 Historical demographic trends of WE group represented by Bayesian skyline plot (BSP). The time scale before present is shown on the x-axis, and the estimated effective population size appears on the y-axis. Estimates of means are joined by a solid line, and the shaded range delineates the 95% HPD limits.

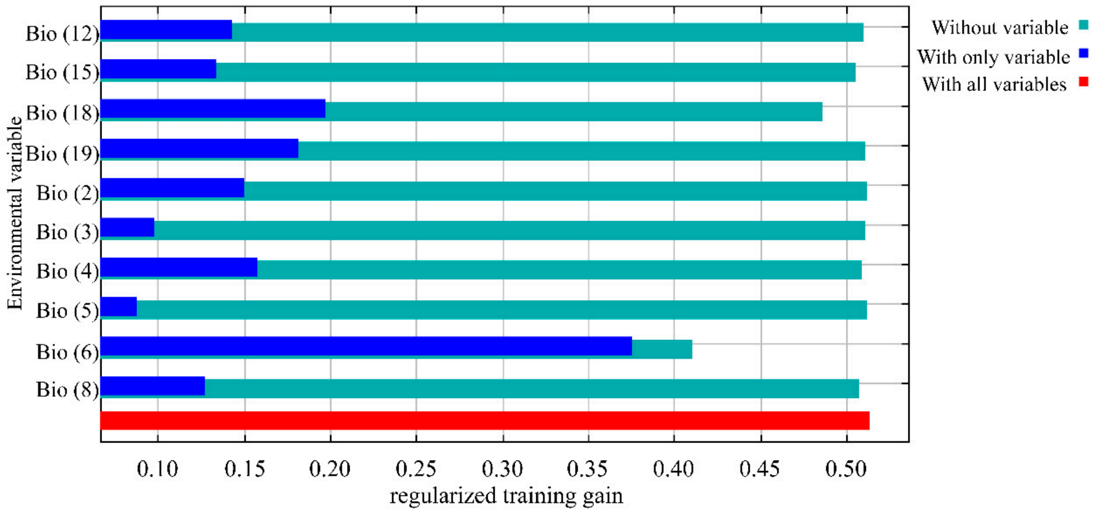

Figure S6 Jackknife of regularized training gain of environmental variables for *Chauliops fallax*.

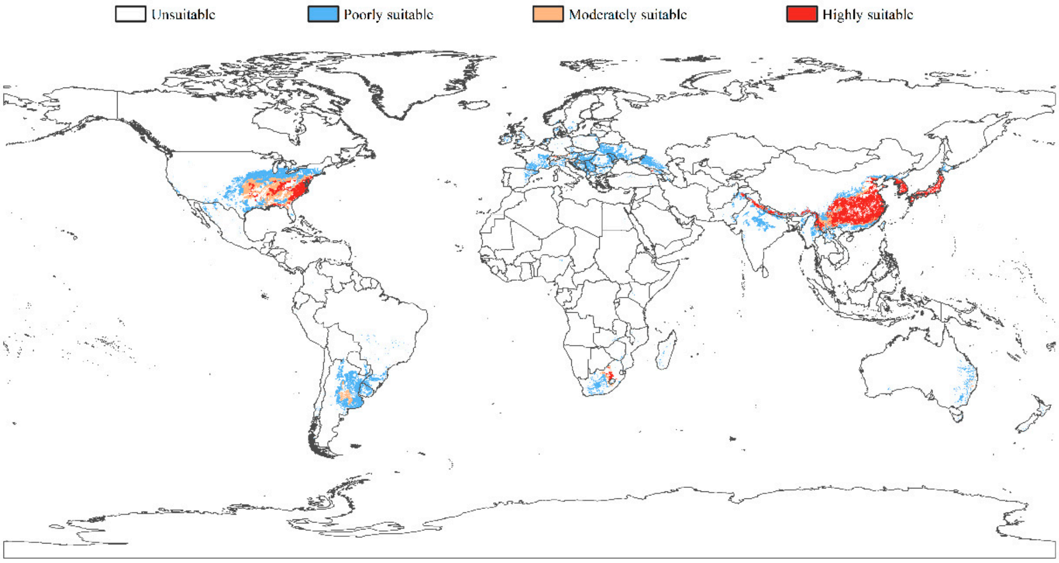

Figure S7 The overlapped map between global potential distribution of *Chauliops fallax* and world soybean cropland.

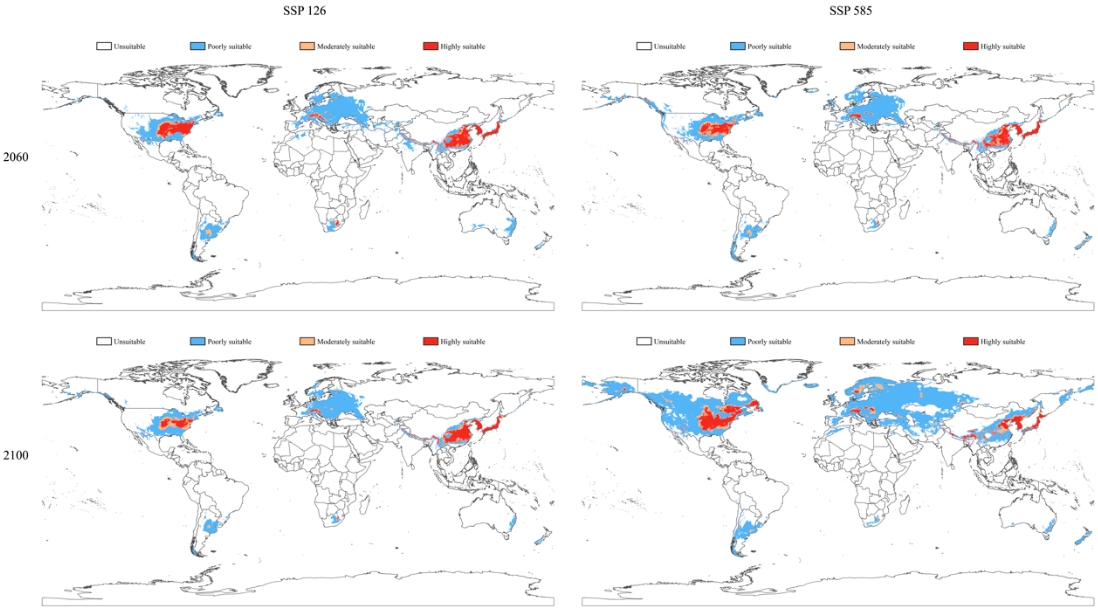

Figure S8 Global potential distribution of *Chauliops fallax* predicted by MaxEnt model at future IPSL-CM6A-LR climate conditions

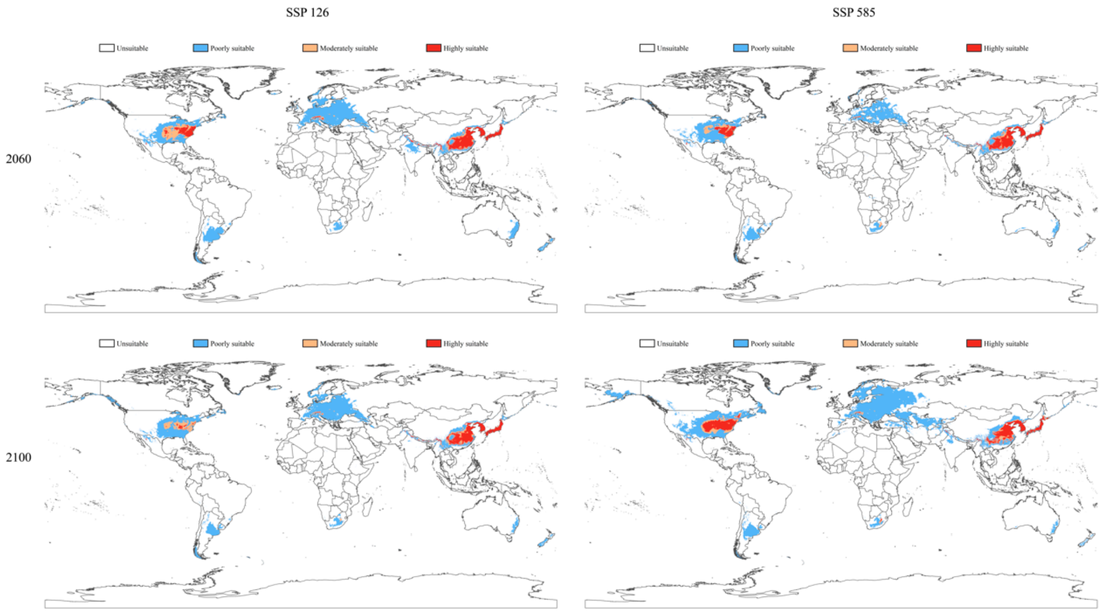

Figure S9 Global potential distribution of *Chauliops fallax* predicted by MaxEnt model at future MPI-ESM1-2-LR climate conditions
